# Neuritin can normalize neural deficits of Alzheimer's disease

**DOI:** 10.1038/cddis.2014.478

**Published:** 2014-11-13

**Authors:** K An, J H Jung, A Y Jeong, H G Kim, S Y Jung, K Lee, H J Kim, S-J Kim, T-Y Jeong, Y Son, H-S Kim, J-H Kim

**Affiliations:** 1 Department of Life Sciences, Pohang University of Science and Technology (POSTECH), Pohang, Gyungbuk 790-784, Korea; 2 Department of Pharmacology, College of Medicine, Seoul National University, Seoul 110-799, Korea

## Abstract

Reductions in hippocampal neurite complexity and synaptic plasticity are believed to contribute to the progressive impairment in episodic memory and the mild cognitive decline that occur particularly in the early stages of Alzheimer's disease (AD). Despite the functional and therapeutic importance for patients with AD, intervention to rescue or normalize dendritic elaboration and synaptic plasticity is scarcely provided. Here we show that overexpression of *neuritin*, an activity-dependent protein, promoted neurite outgrowth and maturation of synapses in parallel with enhanced basal synaptic transmission in cultured hippocampal neurons. Importantly, exogenous application of recombinant neuritin fully restored dendritic complexity as well as spine density in hippocampal neurons prepared from Tg2576 mice, whereas it did not affect neurite branching of neurons from their wild-type littermates. We also showed that soluble recombinant neuritin, when chronically infused into the brains of Tg2576 mice, normalized synaptic plasticity in acute hippocampal slices, leading to intact long-term potentiation. By revealing the protective actions of soluble neuritin against AD-related neural defects, we provide a potential therapeutic approach for patients with AD.

Efficient neuronal communications through synapses are crucial for normal brain functions, whereas alterations in synapse numbers, dendritic spine morphology, and dendritic complexity are thought to be reflected by different forms of synaptic plasticity and are also causally associated with a variety of neurological disorders.^1, 2, 3, 4, 5^ For example, synapse loss and neurite atrophy are the major neurobiological substrates underlying memory impairment in neurodegenerative diseases such as Alzheimer's disease (AD).^6, 7^ The increased dendritic mislocalization of hyperphosphorylated tau protein, a microtubule-associated protein enriched at axons of mature neurons,^8^ and abundance of soluble oligomeric forms of *β*-amyloid (A*β*) appear to cause the synaptic defects and disruption of synaptic plasticity involving the progression of AD pathology.^6, 9, 10^ The apparent decreases in neurotrophic factors observed in brains of patients with AD^11^ have prompted several trials for administration of neurotrophic factors, such as brain-derived neurotrophic factor (BDNF), to attenuate and possibly reverse synaptic defects.^11, 12, 13^ However, the truncation or decreased expression of its cognate receptors in AD brains have limited their potential usage as AD therapeutics.^12, 14, 15^

Neuritin, also known as the candidate plasticity gene 15, was originally identified in a screening study for activity-regulated genes and was subsequently found to be one of the signaling molecules downstream to BDNF and its receptor tropomyosin-related kinase receptor type B.^16, 17^ Ensuing studies indicated that neuritin could also be induced by experimental seizure or by normal life experiences, such as sensory stimulation and exercise.^17, 18, 19, 20, 21, 22^ Located in the 6p24-p25 interval on chromosome 6,^23^ the neuritin gene encodes a small, highly conserved protein containing a secretory signal sequence at the N-terminus and a consensus sequence for glycosylphosphatidylinositol (GPI) at the C-terminus.^16^ This GPI linkage enables neuritin to anchor at cell surfaces, and upon cleavage of GPI by phospholipase the resultant soluble neuritin is released into the extracellular space.^16, 20, 24, 25, 26^

During embryonic neural development, neuritin is mainly expressed in brain regions that undergo a rapid proliferation of neuronal progenitor pools, suggesting a protective role of neuritin for differentiated neurons.^26, 27^ Interestingly, the expression level of neuritin remains elevated after birth or even increases, especially in brain regions presumably exhibiting high neural activity and synaptic plasticity, such as the hippocampus, visual cortex, and external granular layer of the cerebellum.^16, 19, 20, 26^ In addition, neuritin promotes neuritic arbor growth and synaptic formation.^16, 20, 24, 25, 28, 29, 30, 31^ Although various studies have suggested these potent neuritogenetic activities of neuritin, the contribution of neuritin expression to or its effectiveness against neurodegenerative diseases that display neurite atrophy and synapse loss has been largely unexplored.

Here we determined that neuritin expression increased neurite complexity and promoted the maturation of individual spines in cultured hippocampal neurons. Consistent with these findings, basal synaptic transmission was enhanced by transient expression of neuritin. Importantly, when exogenously applied, the soluble neuritin peptide rescued the dendrite complexity of neurons prepared from Tg2576 mice, a transgenic mouse model of AD, such that the complexity was comparable to that in wild-type (WT) mice and also normalized synaptic plasticity in the hippocampus of the Tg2576 mice. Taken together, these results suggest that neuritin, particularly a soluble form of neuritin, reverses synaptic defects manifest in Tg2576 mice and that manipulations to increase neuritin levels may be beneficial therapeutic approaches in AD.

## Results

### Neuritin increases neurite outgrowth

**Figure 1:**
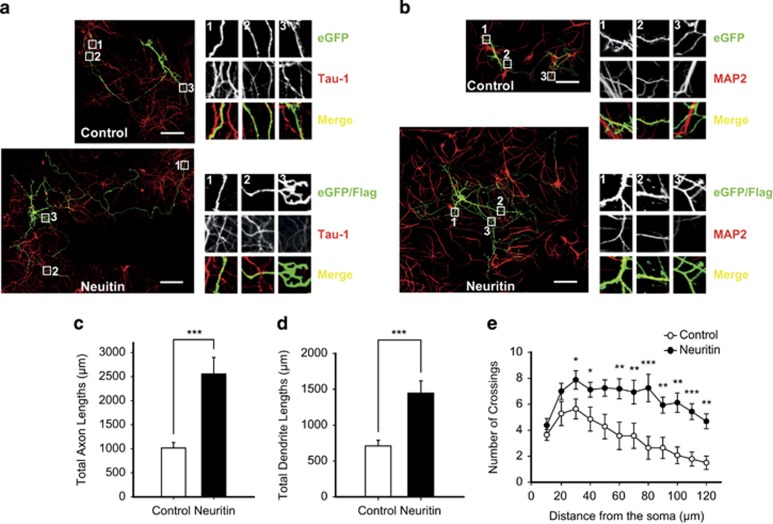
Neuritin expression increases neurite outgrowth. (**a** and **b)** Confocal images of either control vector- or neuritin-expressing neurons that were co-immunostained with eGFP alone or eGFP/Flag (green), Tau-1 (**a**, red) or MAP2 (**b**, red). Scale bar, 100 nm. Insets are × 6.9 magnified views of designated regions. Total axonal length (**c**: Control, 1017.13±112.96 *μ*m, *n*=19 neurons *versus* Neuritin, 2555.57±341.08 *μ*m, *n*=26 neurons) and dendritic length (**d**: Control, 709.35±78.79 *μ*m, *n*=23 neurons *versus* Neuritin, 1446.69±171.17 *μ*m, *n*=30 neurons) of control- and neuritin-expressing neurons are depicted. (**e**) Sholl analyses for neurites visualized with Flag staining. Neuritin slowed down the decrement rate of crossing numbers (at 30 *μ*m: Control, 5.64±0.76 *versus* Neuritin, 7.88±0.71, *P=0.0402; at 40 *μ*m: 4.86±0.93 *versus* 7.13±0.58, *P=0.0415; at 60 *μ*m: 3.57±1.06 *versus* 7.19±0.77, **P=0.0011; at 70 *μ*m: 3.57±0.97 *versus* 6.94±0.91, **P=0.0053; at 80 *μ*m: 2.64±0.88 *versus* 4.25±1.06, ***P=0.0004; at 90 *μ*m: 2.64±0.82 *versus* 5.94±0.62, **P=0.0018; at 100 *μ*m: 2.27±0.63 *versus* 5.71±0.68, **P=0.0018; at 110 *μ*m: 1.79±0.55 *versus* 5.44±0.61, ***P=0.0009; at 120 *μ*m: 1.91±0.51 *versus* 4.69±0.58, **P=0.0012). Statistical significance is expressed as *P<0.05; **P<0.01; ***P<0.001

Neuritogenesis is a key cellular process that sculpts neural circuits by controlling axonal arbors and dendritic branching during neural development, and also contributes to the remodeling of neural connectivity underlying synaptic plasticity in adulthood.^32^ This process is controlled not only by cell-intrinsic factors but also by extrinsic factors involved in synaptic activity.^32^ Neuritin has been shown to be a potent regulator for neuritogenesis *in vitro* and *in vivo*.^16, 24, 26^ To validate the neuritogenetic effect of neuritin in cultured neurons, we transfected neurons with full-length neuritin (pIRES-eGFP-neuritin-Flag)^26^ or its control vector. As the outgrowth of neurites starts early in postnatal development,^29, 33^ hippocampal neurons in culture were transfected at 3 days *in vitro* (DIV) and analyzed 3 days later. Those transfected neurons were identified with the expression of eGFP or neuritin-Flag that was labeled with Alexa Fluor 488, because the intensity of the eGFP signal itself from pIRES-eGFP-neuritin-Flag tended to be weak. Axonal and dendritic processes were discriminated with Tau-1 and microtubule-associated protein 2 (MAP2), respectively (Figures 1a and b).^8^ A series of confocal images was acquired and reassembled to elucidate entire neuronal structures. Compared with neurons transfected with the control vector, neuritin expression for 3 days substantially increased both total axonal (***P<0.001, Mann–Whitney *U-*test; Figures 1a and c) and total dendritic lengths (***P=0.0005, Mann–Whitney *U-*test; Figures 1b and d).

Neurite complexity is also important for neuronal activity through neural circuits, because the complexity affects action potential propagation as well as intrinsic firing.^34, 35^ Thus, we counted the number of neuritic intersections for concentric circles with radii increasing by 10 *μ*m (Sholl analysis)^36^ to quantitatively analyze the complexity of the neuritic trees. In control vector-transfected neurons, intersection numbers peaked at 30 *μ*m from the soma and then decreased continuously (Figure 1e). As observed in the optic tectal neurons or motor neurons of *Xenopus laevis*,^24, 25, 28^ neuritin increased the number of intersections by ~40% at the peak and then attenuated the rate of decrement (Figure 1e). These results clearly indicated that the transient expression of neuritin promoted outgrowth of both axonal and dendritic processes and dramatically increased the complexity of the neuritic trees. Although we assessed the neuritogenetic activity of neuritin at an early development stage (6 DIV), the regulated expression of neuritin may modulate neuritic elaboration and, in turn, may affect the quality and quantity of synaptic transmission even at later stages.

### Neuritin promotes synaptic maturation

**Figure 2:**
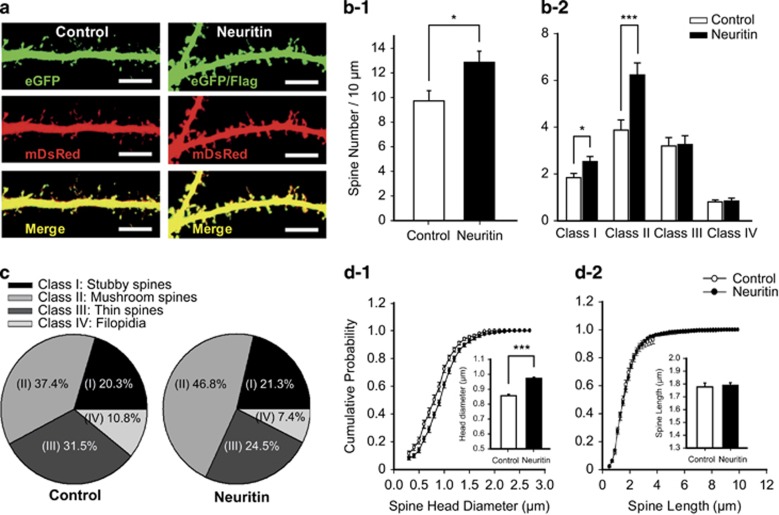
Neuritin expression enhances maturation of spines. (**a**) Representative images of either control vector (left)- or neuritin (right)-expressing neurons immunostained with eGFP/Flag and (green, top) and simultaneously labeled monomeric DsRed (mDsRed, red, middle). Fluorescence images are merged (bottom). Scale bar, 10 *μ*m. (**b**) Spine numbers per 10 *μ*m are measured (**b–1**: Control, 9.73±0.83 *versus* Neuritin, 12.86±0.90) and categorized into their subtypes (**b–2**: Class I: Control, 1.85±0.18 *versus* Neuritin, 2.53±0.22; Class II: 3.88±0.43 *versus* 6.23±0.51; Class III: 3.20±0.36 *versus* 3.26±0.38; Class IV: 0.80±0.09 *versus* 0.84±0.12) from control vector- (*n*=31) and neuritin-expressing (*n*=34) neurons. (**c**) Pie charts showing the frequency in which each type of spines are observed. (**d**) Cumulative probability diagrams for spine head diameters (**d–1**) and spine lengths (**d–2**). Insets: averaged spine head diameters (Control, 0.86±0.1 *μ*m *versus* Neuritin, 0.97±0.01 *μ*m) or spine lengths (1.78±0.03 *μ*m *versus* 1.79±0.02 *μ*m) are depicted, respectively. For (**c** and **d**), 1345 spines from 31 control vector-expressing neurons and 2245 spines from 34 neuritin-expressing neurons are used. Statistical significance is expressed as *P<0.05 and ***P<0.001

Neuritin was shown to enhance dendritic elaboration and synaptogenesis,^27, 30, 31^ but whether neuritin expression has an impact on the maturation of mammalian synapses remains unknown. Thus, we analyzed the morphology of individual spines as well as spine density after transfection at 9 DIV and an additional incubation of 14 days. Co-transfection of monomeric DsRed enabled us to visualize and quantitatively analyze dendritic spine structures independent of neuritin expression. Neuritin-expressing neurons exhibited an increase in spine density compared with control neurons (*P=0.0134, unpaired *t*-test; Figures 2a and b–1).

The shape of a dendritic spine is markedly changed by the synaptic activity imposed on it, and thus the structural signature may represent synaptic efficacy.^2, 3, 4, 37^ Spines were categorized into four classes based on spine head diameter (width) and length, encompassing head and neck: I–IV: stubby, mushroom, thin, and filopodia, respectively.^3, 37, 38^ Each class of spine has a distinct function in synaptic transmission and contributes differently to synaptic plasticity; mushroom spines tend to be more functionally competent than the others.^5^ When we classified individual spines (see the Materials and Methods for detailed criteria),^38^ neuritin-expressing neurons displayed a significantly increased number of spines belonging to class I (*P=0.0269, Mann–Whitney *U-*test) and class II (***P<0.001) compared with control. By contrast, the expression of neuritin did not affect the numbers of spines in class III (*P*=0.8696) or class IV (*P*=0.7775) (Figures 2b–2). Accordingly, out of the total number of protrusions, the proportion of class II spines was increased by ~10%, with slight decreases in the proportions of class III and class IV spines (Figure 2c). Supporting the increased proportion of the class II spines, neuritin expression resulted in an increase in mean diameter of total spine heads (***P<0.001, Kolmogorov–Smirnov test; Figures 2d–1) but did not affect spine length (*P*=0.1344, Kolmogorov–Smirnov test; Figures 2d–2). Consistent with our data, mushroom spines (class II) on neurons in the visual cortex were less abundant in mice lacking neuritin than those in their WT littermates.^29^ Therefore, neuritin likely has a role in stabilizing functionally mature synapses, or possibly in facilitating the maturation of dendritic spines.

### Neuritin enhances basal synaptic transmission

**Figure 3:**
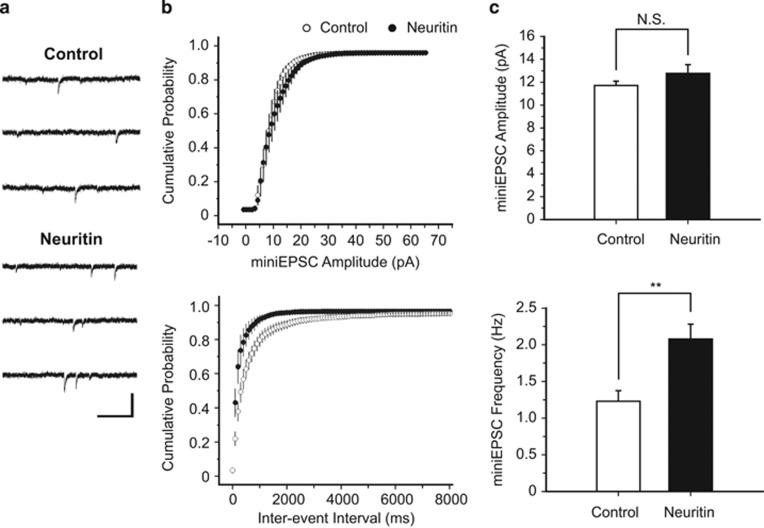
Neuritin expression increases miniEPSC frequency in cultured hippocampal neurons. (**a**) Representative traces of miniEPSCs recorded from control vector (top)- or neuritin (bottom)-expressing hippocampal neurons. Calibration: 20 pA and 200 ms. (**b**) Cumulative probability diagrams of miniEPSC amplitudes (top) and inter-event intervals (bottom). (**c**) Mean miniEPSC amplitude (top: Control, 11.7±0.4 pA *versus* Neuritin, 12.7±0.7 pA) and miniEPSC frequency (bottom: 1.2±0.2 Hz *versus* 2.08±0.2 Hz) are compared between control vector- (*n*=10) and neuritin- (*n*=13) expressing neurons. Statistical significance is expressed as **P<0.01

The promotion of neuritic outgrowth and dendritic spine maturation suggested that neuritin may control the functionality of synaptic connections. As in the morphological analyses of spines, hippocampal neurons were transfected with either control or neuritin vectors at 9 DIV and incubated for an additional 14 days. To measure quantal synaptic responses, we recorded miniature excitatory postsynaptic currents (miniEPSCs), mainly *α*-amino-3-hydroxy-5-methyl-4-isoxazolepropionic acid (AMPA) receptor-mediated miniEPSCs, at a holding potential of −70 mV (Figure 3a). The amplitude of miniEPSCs represent the quantal size normally derived from vesicular transmitter content or the postsynaptic AMPA receptor abundance, whereas changes in the frequency of miniEPSCs signify alterations in the release probability for the presynaptic vesicles or in the number of synapses containing AMPA receptors.^39^ Importantly, neuritin significantly increased the frequency of miniEPSCs compared with that in controls (**P=0.007, Mann–Whitney *U-*test; Figures 3b and c) but did not affect the amplitude of miniEPSCs (*P*=0.3, Mann–Whitney *U-*test; Figures 3b and c). An increase in the frequency of miniEPSC was also observed in the optic tectal neurons of *X. laevis* that virally expressed neuritin.^25^ Therefore, these data indicate that neuritin increases the number of functional synapses, possibly by promoting the maturation of synapses.

### Soluble neuritin prevents dendritic atrophy in hippocampal neurons from Tg2576 mice

**Figure 4:**
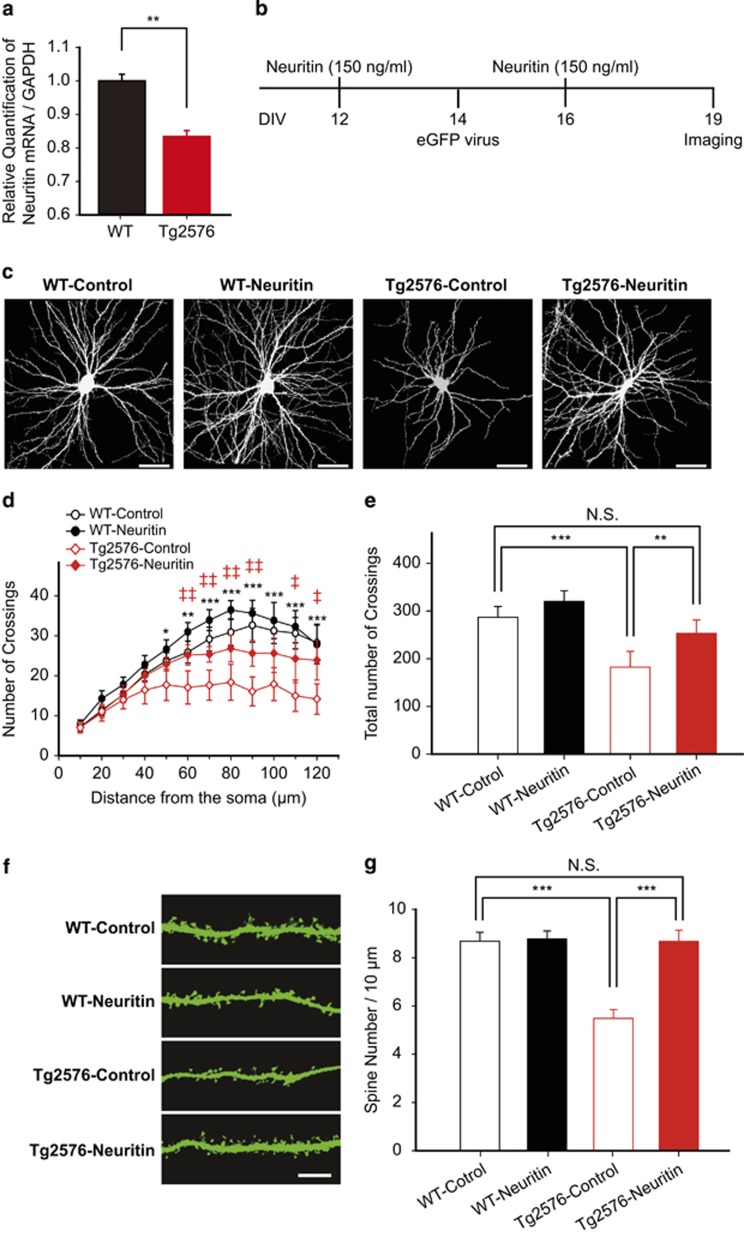
Soluble neuritin peptide prevents neuritic atrophy in neurons from Tg2576 mice. (**a**) mRNA levels of neuritin are depicted from the hippocampi of 6-month-old Tg2576 and WT mice after the normalization to those of the WT littermates (WT, 1±0.0201 *versus* Tg2576, 0.8340±0.0177, *n*=5 mice, respectively). (**b**) The experimental scheme for neuritin peptide treatment and imaging. (**c**) Representative images of eGFP-expressing hippocampal neurons prepared from Tg2576 or their WT littermate mice after treatment of recombinant neuritin peptide (150 ng/ml^16, 27, 42^) or vehicle (PBS as Control). Scale bar, 50 *μ*m. (**d**) Sholl analyses are performed to measure dendritic branch crossings with the designated distanced circles from the soma. Statistical significance between WT-Control *versus* Tg2576-Control is expressed as *P<0.05, **P<0.01, ***P<0.001, whereas the comparison between Tg2576-Control *versus* Tg2576-Neuritin is expressed as ^‡^*P*<0.05, ^‡‡^*P*<0.01. (**e**) Total number of crossings until the marginal branch, ~120 *μ*m is depicted for each group. WT-Control, 286.8±11.3 *versus* WT-Neuritin, 320.4±11.1 *versus* Tg2576-Control, 182.3±16.6 *versus* Tg2576-Neuritin, 253.3±14.1, *n*=11 neurons, respectively. (**f**) Representative images of eGFP-labeled dendrites of Tg2576 and WT neurons after treatment of recombinant neuritin or vehicle (PBS as Control) are presented. Scale bar, 10 *μ*m. (**g**) Total number of spines per 10 *μ*m from each group. WT-Control, 8.69±0.37 *versus* WT-Neuritin, 8.78±0.33 *versus* Tg2576-Control, 5.48±0.37 *versus* Tg2576-Neuritin, 8.68±0.46, *n*=11 neurons, respectively. Multiple comparisons using *post hoc* Bonferroni test after ANOVA reveals a marked decrease in total number of crossings (**e**) and spine numbers (**g**) from Tg2576 neurons compared with those from other groups. Statistical significance is expressed as **P<0.01 and ***P<0.001

The non-cell autonomous function exerted by neuritin suggests that it acts as a ligand at unknown receptors.^24, 25^ In fact, neuritin was shown to exist predominantly as a soluble secreted form *in vivo*,^26^ and several studies indicated that this soluble form had neurotrophic effects in mammalian neurons.^16, 26, 27^ Neuritic atrophy is typically observed in both the brains of patients with AD and models of AD,^40^ and it is readily recapitulated in cultured neurons prepared from Tg2576 mice.^41^ To elaborate potential involvement of neuritin in neuritic atrophy, we measured mRNA level of neuritin in the hippocampi of 6-month-old Tg2576 mice, which revealed a decrease in neuritin mRNA when compared with that of WT littermate control (**P=0.0079, unpaired *t*-test; Figure 4a). Thus, we decided to examine whether the increase of neuritin offers a protective action for the dendritic elaboration in cultured hippocampal neurons from Tg2576 mice or from their WT littermates. In order to properly control the concentration of neuritin throughout the following experiments, we exogenously applied recombinant neuritin peptide, a soluble neuritin at 150 ng/ml,^16, 27, 42^ rather than overexpressing full-length neuritin. For visualization of entire dendritic branches, we used rabies virus encoding eGFP (SADΔG eGFP)^43^ and analyzed both the dendritic arborization using Sholl analysis and spine density following the neuritin treatment (Figure 4b). Unexpectedly, the soluble neuritin peptide alone did not affect the dendritic arborization in WT control neurons at any distance from the soma (Figures 4c–e and Supplementary Table 1), which differed from our results with the transient expression of full-length neuritin (Figure 1).^24, 25, 28, 30^ Spine numbers also were not changed by the treatment of neuritin peptide (*P*>0.05, one-way ANOVA with *post hoc* Bonferroni; Figures 4f and g).

Consistent with a previous report,^41^ neurons isolated from Tg2576 mice displayed an oversimplification in their dendritic trees, as shown by the significantly decreased numbers of total dendritic crossings (***P<0.001, one-way ANOVA with *post hoc* Bonferroni; Figure 4e) and spines (***P<0.001; Figures 4f and g) compared with those from WT control mice. Importantly, treating neurons from Tg2576 mice with soluble neuritin peptide markedly attenuated the decrease in dendritic elaboration (Tg2576-Neuritin *versus* Tg2576-Control, **P=0.0036; Figure 4e) and synaptic formation (***P<0.001; Figure 4g), and even reversed it nearly to the level of that observed in WT neurons (*P*>0.05 in both dendritic crossings and spine numbers; Figures 4e and g, respectively), consistent with our recent observation.^44^ To further examine the effectiveness of neuritin, we treated Tg2576 neurons with increasing concentrations of soluble neuritin. The complexity of neuritic processes of Tg2576 neurons became enhanced in a dose-dependent manner to used neuritin peptide and was comparable to the control level particularly from the 150 ng/ml (Supplementary Figure 1). Therefore, soluble neuritin likely has a protective role against the dendritic degeneration manifested in models of AD and possibly in patients with AD.

### Soluble neuritin rescues hippocampal synaptic plasticity in Tg2576 mice

A number of animal models of AD exhibit deficits in the synaptic plasticity of hippocampal circuits, primarily due to the synaptotoxic activities of oligomeric A*β*.^45, 46, 47, 48^ For instance, long-term potentiation (LTP), an electrophysiological representative of synaptic strengthening in the neuronal connections underlying learning and memory,^49^ in the hippocampus of Tg2576 mice is severely impaired at 6 months of age concomitant with a steep increase in toxic A*β* peptide and memory deficits.^50, 51, 52, 53^ Given the observed neuroprotective role of soluble neuritin as well as a recent report that neuritin ameliorated cognitive abilities in Tg2576 mice,^44^ we reasoned that soluble neuritin may have an impact on LTP in Tg2576 mice.

**Figure 5:**
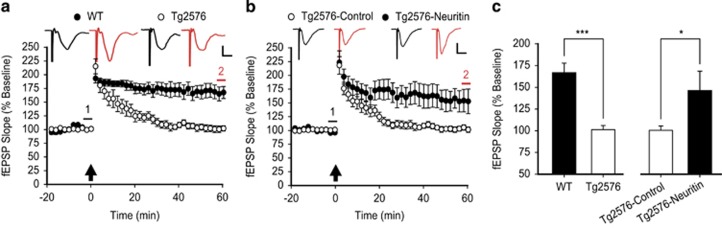
Soluble neuritin rescues LTP in the hippocampi of Tg2576 mice. (**a**) LTP in the Schaffer collateral pathway of 6-month-old WT littermates and Tg2576 mice. Field EPSPs were recorded from the CA1 region of acute hippocampal slices and LTP was induced by TBS, denoted as a black arrow. LTP is expressed as mean±S.E.M.% of baseline slopes of fEPSPs recorded over at least a 20-min baseline period. Insets show representative traces at the color-matched time points (Calibration: 1 mV and 10 ms). (**b**) LTP of Tg2576 mice that underwent the osmotic infusion of either aCSF or recombinant neuritin peptide. Arrow, insets, and calibration as in **a**. (**c**) A summary histogram for the synaptic potentiation at 1 h after LTP induction in **a** and **b**. The magnitude of LTP in Tg2576 mice significantly decreased (WT, 166.8±10.9%, *n*=8 slices from 3 mice *versus* Tg2576, 101.4 ±4.8%, *n*=9 slices from 3 mice), but resumed by osmotic infusion of soluble neuritin peptide (Tg2576-Control, 100.5±4.9%, *n*=12 slices from 4 mice *versus* Tg2576-Neuritin, 146.3±22.2, *n*=9 slices from 4 mice). Statistical significance is expressed as *P<0.05 and ***P<0.001

Initially, we compared LTP in the Schaffer collateral pathway of acute hippocampal slices from 6-month-old Tg2576 mice with that in their WT littermates. Consistent with previous reports,^47, 50^ LTP was significantly impaired in Tg2576 mice when compared with WT mice (***P=0.00004, unpaired *t*-test; Figures 5a and c). To address the possibility that soluble neuritin affected this LTP deficit, we infused recombinant neuritin peptide into the cerebroventricles of 6-month-old Tg2576 mice using osmotic pumps for 2 weeks and then examined LTP. We found that this chronic infusion of soluble neuritin into the brains of Tg2576 mice rescued LTP, whereas Tg2576 mice that had received artificial cerebrospinal fluid (aCSF) alone still exhibited impairments in LTP (*P=0.03346; Figures 5b and c). Indeed, the magnitude of the LTP elicited from the Tg2576 mice infused with soluble neuritin was comparable to that from WT mice (Tg2576-Neuritin *versus* WT, *P*=0.438; Figure 5c). Taken together, these results indicate that soluble neuritin functionally restores synaptic plasticity, likely by its neuroprotective activity at the circuit level.

## Discussion

The functional roles of neuritin in the developing nervous system have been studied extensively, whereas the physiological actions of neuritin on synaptic features, particularly those associated with neurodegenerative diseases, remain largely unknown. Here we assessed the promoting effects of neuritin on neuritic elaboration, dendritic spine maturation, and synaptic transmission. We demonstrated for the first time that soluble neuritin reversed neuritic atrophy and the impairment in synaptic plasticity manifested in the Tg2576 transgenic mouse model of AD, supporting a role for non-cell autonomous functions of neuritin.

### Neurotrophic roles of neuritin

The term ‘neurotrophic' is generally used to describe a collection of effects that triggers neuritogenesis, branch arborization, synaptogenesis, or survival of differentiating neurons.^11^ Previous studies using transient expression of the neuritin gene or application of neuritin peptide provided evidence that neuritin exerts neurotrophic roles at least during developmental stages. These neurotrophic functions include the promotion of neuritogenesis,^16, 24, 27^ regulation of synapse formation and stabilization,^25, 28, 29, 31^ and prevention of programmed cell death.^26, 27^ Consistent with these neurotrophic actions, the expression pattern of neuritin is spatiotemporally correlated with the expansion of neural progenitor cells^26, 54^ and activity-induced synaptic modifications.^18, 19, 20, 22^ For example, neuritin transcription in the visual cortex is enhanced during eye opening,^19, 55^ when marked increases also occur in dendritic branching complexity and spine numbers, as well as in miniEPSCs.^33, 56, 57^

We extended previous findings to the action of neuritin on the formation and maturation of dendritic spines, showing that the expression of neuritin resulted in increases in the numbers and proportions of stubby- and mushroom-type spines. In contrast to the versatile small spines (filopodia and thin spines) that usually form silent or weakly glutamate-sensitive synapses, large spines (stubby and mushroom spines) with large postsynaptic densities are highly enriched with AMPA receptors and are thereby potently sensitive to presynaptic glutamate release.^2, 58^ The expression of neuritin resulted in an increase in the frequency but not the amplitude of miniEPSCs. Combined with the increased abundance of functionally mature spines, the selective impact on the frequency of miniEPSCs may be attributable to the neuritin-mediated conversion of silent synapses into functional synapses, involving incorporation of AMPA receptors into pure-N-methyl-D-aspartate (NMDA) receptor synapses.^25^ Alternatively, postsynaptic expression of neuritin may alter presynaptic features, such as an increase in the release probability of presynaptic vesicles, in a retrograde manner, which results in an increased frequency of miniEPSCs.^39^ A detailed analysis of the molecular mechanisms underlying the synaptic effects of neuritin will contribute to further understanding of the functional maturation of synapses.

### Full-length neuritin *versus* soluble secreted neuritin

Similar to endogenous neuritin, exogenously expressed neuritin exists in both cell surface-attached and secreted forms.^26^ In the optic tectal neurons of *X. laevis*, expression of full-length neuritin promoted axonal outgrowth or dendritic branching, as well as conversion of silent synapses into functional synapses, whereas the truncated form of neuritin lacking the GPI sequence did not.^24, 25^ Intriguingly, the secreted form of neuritin including neuritin peptide resulted in neurotrophic consequences in mammalian neurons, including the promotion of neuritic elaboration and prevention of apoptosis.^16, 26, 27^ There is a possibility that the GPI-lacking neuritin might be improperly processed and thereby could not be secreted to the extracellular space.^59^ However, it should be determined whether and how the soluble neuritin produces distinct outputs compared with cell surface-attached neuritin.

Surprisingly, the effect on dendritic complexity of soluble neuritin applied to neurons from WT mice was undistinguishable from that of PBS-treated neurons (Figure 4). This lack of effect for soluble neuritin was in stark contrast with that which had been previously observed in cortical and hippocampal neurons.^16, 26, 27^ This discrepancy may be attributable to the difference in neuronal developmental stage, that is, DIV. We examined dendritic complexity at a late stage (DIV, 19) after application of soluble recombinant neuritin, whereas the analyses in those previous studies were conducted at earlier stages (DIV, 3–7).^16, 26, 27^ This possibility is further supported by our observation that neuritic elaboration was increased by neuritin expression assessed at 6 DIV (Figure 1). The different morphological effectiveness of neuritin within a limited time window may be due to a ceiling effect for the signaling mechanisms activated by endogenous neuritin, as the level of endogenous hippocampal neuritin increased from postnatal day 4 to adulthood.^16^ Recapitulating the increase in endogenous neuritin in cultured hippocampal neurons may intensify the downstream signaling cascades, which are fully saturated at 12 DIV; thus, exogenous application of soluble neuritin thereafter would not produce additional effects. Negligible effect of neuritin peptide on matured WT neurons (Figure 4) prompts us to surmise that neuritin has roles in dendritic arborization, synapse formation, and synaptic transmission during the early stages of development, but not when its expression saturates at the later stage (DIV, 19). Given a decreased level of neuritin in the brains of patients with AD^44^ and in the hippocampi of Tg2576 mice (Figure 4a), it is possible to speculate that exogenous neuritin may counteract the reduction of endogenous neuritin observed in neurons from Tg2576 mice to reverse neuritic atrophy.

### Soluble neuritin prevents synaptic deficits in AD

Importantly, we showed that soluble neuritin prevented the dendritic atrophy and LTP impairment in hippocampal neurons and the hippocampi, respectively, from Tg2576 mice. Both deficits involve caspase-3 activation.^46, 60, 61^ Indeed, synaptotoxic A*β* was shown to activate caspase-3, resulting in cleavage of Akt1 (also known as protein kinase B-*α*), the activity of which is critically required for dendritic arborization and synaptic plasticity.^46, 60, 62, 63^ Hence, it is conceivable that the activation of a putative receptor for neuritin interferes with the activation of caspase-3 (Putz *et al.*^26^) and subsequent cleavage of Akt1, although this possibility has not been assessed.

Recently, it was reported that soluble neuritin triggered the insulin receptor pathway, suggesting the insulin receptor as a putative receptor for neuritin.^42^ In addition, soluble neuritin stimulated extracellular signal-regulated kinase (ERK) and the mammalian target of rapamycin (mTOR) via the insulin receptor.^42^ Activities of ERK and mTOR were intimately involved in *de novo* protein synthesis, a prerequisite to normal synaptic plasticity and memory formation.^49, 64, 65^ Emerging evidence indicates that the dysregulation of mTOR signaling causally contributes to the pathogenesis of AD, although this remains debatable.^64, 66, 67, 68^ In Tg2576 mice, mTOR activity was substantially suppressed, and pharmacological upregulation of mTOR rescued LTP.^66^ The functional significance of mTOR in the synaptic defects of AD was corroborated by behavioral data from another animal model of AD.^68^ Therefore, the normalization of LTP in Tg2576 mice by infusion of soluble neuritin may be due to a neuritin-mediated increase in mTOR activity. Given the neuroprotective activities of soluble neuritin, especially for neurons from Tg2576 mice rather than those from WT controls, it will be informative to determine whether the infusion of soluble neuritin into the brains of Tg2576 mice also normalizes their memory deficits.

In summary, neuritin treatment alone yielded neurotrophic activities in neurite outgrowth and dendritic spine maturation in normal neurons, particularly in early developmental stages. Importantly, we demonstrated that soluble neuritin executed neuroprotective actions for neurons and the hippocampal circuits in Tg2576 mice, which prevented dendritic atrophy and impairment in synaptic plasticity. We provided substantial evidence that soluble neuritin reverses the synaptic defects in an animal model of AD, suggesting that manipulating the level of endogenous neuritin or supplying exogenous soluble neuritin may offer therapeutic benefits against neurodegenerative diseases.

## Materials and Methods

### DNA and viral constructs

We used pIRES-EGFP-neuritin1-FLAG for overexpression of neuritin and pIRES-EGFP for its control plasmid on primary hippocampal neurons, which were kindly provided by Dr Nedivi in MIT.^26^ To visualize or categorize dendritic spines, we used pDsRed-Monomer-N1 for additional transfection or rabies virus encoding enhanced GFP (SADΔG eGFP) for viral infection.^43^

### Hippocampal neuron culture, transfection, and immunocytochemistry

Primary hippocampal neurons dissected from embryonic day 18 of C57BL/6 mice were plated on poly-L-lysine (Sigma, St. Louis, MO, USA) coated coverslips. Neurons were maintained in neurobasal medium (Invitrogen, Carlsbad, CA, USA) supplemented with B27 (Invitrogen), 5 mM L-glutamine (Sigma), 2.5 *μ*M cytosine *β*-D-arabinofuranoside (Sigma), 5% fetal bovine serum (Hyclone, Logan, UT, USA), and 1% penicillin/streptomycin (Invitrogen) under a humidified environment of 5% CO_2_/95% O_2_ incubator at 37 °C.

Neurons were transfected with Calcium Phosphate Transfection Kit (Invitrogen) following the previously described methods.^69^ Briefly, we incubated neurons in pre-warmed minimum essential medium (Invitrogen) supplemented with 5 mM MgCl_2_ for 30 min and dissolved the pre-mixed DNA/Calcium Phosphate precipitate to the medium. After 45 min incubation, neurons were washed three times with pre-equilibrated HBSS in 10% CO_2_/90% O_2_ incubator at 37 °C.

For immunocytochemistry, neurons were fixed with PBS containing 4% paraformaldehyde, 4% sucrose for 10 min at 37 °C, and permeabilized with PBS containing 0.2% Triton X-100, 20 mM glycine for 5 min at room temperature. Neurons were washed three times with PBS for 5 min, blocked with 2% bovine serum albumin for 30 min at room temperature, and incubated with primary antibodies against Flag (Cell Signaling Technology, Danvers, MA, USA), MAP2 (Sigma), and Tau-1 (Millipore, Bedford, MA, USA) at 4 °C for overnight. After washing, secondary antibodies conjugated to Alexa Fluor 488 (Invitrogen) or Alexa Fluor 568 (Invitrogen) were further incubated at 37 °C for 45 min.

### Image acquisition and quantitative morphometry

Fluorescence images were acquired using an Olympus Fluoview 1000 confocal microscope. Images collected from three or four independent experiments under the same parameters were analyzed using MetaMorph imaging software (Universal Imaging, Bedford Hills, NY, USA). To avoid dying neurons affecting the result, neurons with vacuoles in the soma and with dendritic fragmentation were excluded from the analysis.

For the dendritic spine analysis, sparsely transfected neurons were chosen to minimize dendritic overlapping and a stack of images was acquired in the *z*-dimension at optical slice thickness of 0.4 *μ*m to cover entire neurons. The criteria for spine categorization were used following the method as described previously.^38^ In brief, protrusions along the dendrites were classified into four classes of spines depending on their head width and neck length. Class I, ‘stubby' for short length <0.5 *μ*m, lacking a large heads without neck; Class II, ‘mushroom' for mature spines with a lengh between 0.5 and 1.25 *μ*m, having a large spine head and a short neck; Class III, ‘thin' for spines with a lengh between 1.25 and 3.0 *μ*m, with a small spine head and a elongated neck; Class IV, ‘fillopodia' for immatuer spines with a long filamentous protrusions lacking any distingtible spine head.

To quantitatively measure the complexity of dendrites, we used Sholl analysis as described previously.^36, 41^ After removing neuritic processes that apparently derived from other cells from the original images, concentric circles with 10 *μ*m interval in radius were drawn 15 *μ*m apart from the soma and the number of crossings with dendritic branches at each circle was counted using Image J (NIH). Sholl analysis was carried out by counting total crossings to a distance ~120 *μ*m from the soma.

### Quantitative real-time RT-PCR

Total RNA was extracted by miRNeasy mini kit (Qiagen, Hilden, Germany) and 0.5 *μ*g of RNA was processed for cDNA synthesis using SuperScript III First-Strand Synthesis System for RT-PCR Kit (Invitrogen) according to the manufacturer's instruction. Quantitative real-time RT-PCR was carried out using SYBR Green PCR Master Mix (Applied Biosystems, Carlsbad, CA, USA). Quantitative real-time RT-PCR was performed on a 7500 Fast Real-Time PCR systems (Applied Biosystems). The primers used were as follows: *Neuritin* forward, 5′-GGGACTTAAGTTGAACGGCA-3′ *Neuritin* reverse, 5′-ACCCAGCTTGAGCAAACAGT-3′ *Gapdh* forward, 5′-TCCATGACAACTT TGGCATTG-3′ *Gapdh* reverse, 5′-CAGTCTTCTGGGT GGCAGTGA-3′. All of the mRNA level were normalized to that of Gapdh mRNA.

### Intracerebroventricular infusion of recombinant neuritin peptide using osmotic pumps

To examine the effect of soluble neuritin on synaptic plasticity in the hippocampus of AD model mice, we used Tg2576 mice that expressed human *APP695* gene harboring the Swedish double mutation (KM670/671NL).^70^ Tg2576 mice (Taconic, Germantown, NY, USA) were crossed with C57BL6/SJL F1 hybrid mice to get the offspring. For LTP experiments, we used 6-month-old heterozygous transgenic mice and their WT littermates. All mice were housed under a 12 h light/dark cycle and given *ad libitum* access to food and water. All procedures for animal experiments were approved by the ethical review committee of POSTECH (Pohang University of Science and Technology), Korea, and performed in accordance with the relevant guidelines.

Installation of the osmotic pumps was performed following the manufacturer's guideline. Forty eight hours before the surgery, the osmotic mini pump (1007D, Alzet, Cupertino, CA, USA) was filled with aCSF (containing the followings: 10 mM glucose, 119 mM NaCl, 2.5 mM KCl, 1.25 mM NaH_2_PO_4_, 1.3 mM MgSO_4_, 2.5 mM CaCl_2_, and 26 mM NaHCO_3_ at pH 7.4) with or without recombinant neuritin peptide (1260 ng, Abcam, Cambridge, UK) and equilibrated in 0.9% NaCl at 37 ^o^C. Delivery cannula (Alzet, brain infusion kit 3) was implanted in order to target the end of cannula to intracerebroventricle (coordination of anteroposterior, −0.4; mediolateral, ±1; dorsoventral, −2.3 in mM from the bregma) of anesthetized (Ketamine/Xylazine) mice in a stereotaxic apparatus (Kopf Instruments, Tujunga, CA, USA). The osmotic pump was attached to the delivery cannula tubing and subcutaneously implanted at the back to allow spontaneous infusion (injection speed: 90 ng/day). After 2 weeks of infusion, animals were killed for the LTP experiment.

### Electrophysiology

To measure miniEPSCs, neurons were placed in a recording chamber while perfused with aCSF containing 1 *μ*M voltage-gated sodium channel blocker tetrodotoxin (Tocris, Minneapolis, MN, USA), 20 *μ*M NMDA receptor antagonist D-2-amino-5-phosphonovaleric acid (Tocris), 100 *μ*M *γ*-aminobutyric acid class A receptor antagonist picrotoxin (PTX, Tocris). Whole-cell voltage-clamp recording was performed with a MultiClamp 700B amplifier (Molecular Devices, Sunnyvale, CA, USA) using the recording electrodes (3–5 MΩ) filled with a pipette solution containing 100 mM Cs methanesulfonate, 20 mM CsCl, 10 mM HEPES, 10 mM EGTA, 4 mM MgCl_2_, 0.4 mM NaGTP, 4 mM MgATP, and 10 mM phosphocreatine (finally adjusted to pH 7.2). Throughout the recording experiments, the series resistance (10–30 MΩ) was monitored while holding neurons at −70 mV. Currents were filtered at 2.4 kHz, digitized at 10 kHz, and then miniEPSCs were analyzed in Mini Analysis Program (Synaptosoft Inc., Fort Lee, NJ, USA) using custom software with detection criteria that included an amplitude>8 pA, a minimum rise rate of 5 pA/ms, and a decay constant between 1–12 ms.

For LTP in acute hippocampal slices, field excitatory postsynaptic potentials (fEPSPs) were recorded from transverse-sectioned acute hippocampal slices (400 *μ*m thick) obtained from 6-month-old male Tg2576 or their WT littermate mice. After decapitation, hippocampi were quickly isolated from the brain and chilled in ice-cold 50% sucrose-based (175 mM sucrose, 11 mM glucose, 20 mM NaCl, 3.5 mM KCl, 1.4 mM NaH_2_PO_4_, 1.3 mM MgCl_2_, and 26 mM NaHCO_3_, adjusted to pH 7.4) aCSF that was oxygenated with 95% O_2_ and 5% CO_2_ gas. Acute hippocampal slices were obtained using an 800-McIlwain Tissue Chopper (Brinkman Instruments, Westbury, NY, USA) and placed in oxygenated aCSF at 37 °C for more than 1 h. Slices were maintained in a submerged recording chamber continuously perfused with oxygenated aCSF bath solution, containing 100 *μ*M PTX, at a flow rate of 2.5–3 ml/min. The fEPSPs were recorded in the striatum radiatum of the CA1 subfield by the 3 M NaCl-filled microelectrodes (3–5 MΩ) while stimulating the Schaffer collateral pathway afferent fibers with a bipolar concentric electrode (WPI). The pulses were generated with an A360 stimulus isolator (WPI) and fEPSPs were recorded with an Axopatch 200A amplifier linked to a Digidata 1200 (Molecular Devices) interface. Test fEPSPs were evoked by the stimulation intensity that yielded one-third of the maximal fEPSP responses at a frequency of 0.033 Hz, and LTP was induced by five episodes of theta burst stimulation (TBS) that were delivered at 0.1 Hz. In each episode, 10 trains of stimulation that consisted of four pulses at 100 Hz were delivered at 5 Hz.

### Statistical analysis

All the numerical data resulted from analysis were denoted as mean±S.E.M.%. Mann–Whitney *U-*test or unpaired *t*-test was used to determine statistical significance between two data set as appropriately. Kolmogorov–Smirnov test was used to compare the spine head diameter and length between groups. In the case of multiple comparisons, one-way ANOVA with *post hoc* Bonferroni test was used. Statistical significance between groups is expressed as follows: N.S., not significant; *P<0.05; **P<0.01; ***P<0.001.

## Abbreviations

AD: Alzheimer's disease
A*β*: *β*-amyloid
BDNF: brain-derived neurotrophic factor
GPI: glycosylphosphatidylinositol
WT: wild type
DIV: days *in vitro*
MAP2: microtubule-associated protein 2
miniEPSCs: miniature excitatory postsynaptic currents
AMPA: *α*-amino-3-hydroxy-5-methyl-4-isoxazolepro-pionic acid
LTP: long-term potentiation
aCSF: artificial cerebrospinal fluid
NMDA: N-methyl-D-aspartate
ERK: extracellular signal-regulated kinase
mTOR: mammalian target of rapamycin
fEPSPs: field excitatory postsynaptic potentials
TBS: theta burst stimulation
