# Supplementary material for: Neuritin can normalize neural deficits of Alzheimer's disease
Source: Cell Death Dis. 2014 Nov 13;5(11):e1523–. doi: 10.1038/cddis.2014.478 (PMC4260736; doi:10.1038/cddis.2014.478)
Supplement: Supplementary Information [file cddis2014478x1.doc]

**Supplemental Information for**

**Neuritin can normalize neural deficits of Alzheimer’s disease**

**Running Title: Neuroprotective roles of neuritin**

Kyongman An, Jung Hoon Jung, A Young Jeong, Hyoun Geun Kim, SangYong Jung, Kihwan Lee, Hyun Jin Kim, Su-Jeong Kim, Tae-Yang Jeong, Yujin Son, Hye-Sun Kim and Joung-Hun Kim

**Supplementary Figure Legend**

**Supplementary Table**

**Supplementary Figure 1. Soluble neuritin peptide normalizes dendritic atrophy in a dose-dependent manner. (a)** Representative images of eGFP-expressing hippocampal neurons prepared from Tg2576 mice after treatment of various concentrations of recombinant neuritin peptide (0–300 ng/ml) and from their WT littermate are presented. Scale bar: 50 μm. (**b**) Representative images of eGFP-labelled dendrites of Tg2576 neurons are presented after treatment of various concentrations of recombinant neuritin as well as WT neurons with vehicle (PBS as Control). Scale bar: 10 μm. (**c**) Total number of crossings until the marginal branch, ~120 μm on (**a**) is depicted for each group. (**d**) Total number of spines per 10 μm dendrites from each group in (**b**). Statistical significance is expressed as **P* < 0.05, ***P* < 0.01 and ****P* < 0.001.

| **Supplementary Table 1. Multiple Comparisons by One-Way ANOVA with Post Hoc Bonferroni Test** | | | | | | | | |
| --- | --- | --- | --- | --- | --- | --- | --- | --- |
| Dependent Variable | (I) Group | (J) Group | Mean Difference (I-J) | Std. Error | Sig. |  | 95% Confidence Interval | |
|  |  |  |  |  |  |  | Lower Bound | Upper Bound |
| Distance010 | WT-Control | WT-Neuritin | -.72727 | .78467 | 1.000 |  | -2.9054 | 1.4509 |
|  |  | Tg-Control | .09091 | .78467 | 1.000 |  | -2.0872 | 2.2690 |
|  |  | Tg-Neuritin | .18182 | .78467 | 1.000 |  | -1.9963 | 2.3599 |
|  | WT-Neuritin | WT-Control | .72727 | .78467 | 1.000 |  | -1.4509 | 2.9054 |
|  |  | Tg-Control | .81818 | .78467 | 1.000 |  | -1.3599 | 2.9963 |
|  |  | Tg-Neuritin | .90909 | .78467 | 1.000 |  | -1.2690 | 3.0872 |
|  | Tg-Control | WT-Control | -.09091 | .78467 | 1.000 |  | -2.2690 | 2.0872 |
|  |  | WT-Neuritin | -.81818 | .78467 | 1.000 |  | -2.9963 | 1.3599 |
|  |  | Tg-Neuritin | .09091 | .78467 | 1.000 |  | -2.0872 | 2.2690 |
|  | Tg-Neuritin | WT-Control | -.18182 | .78467 | 1.000 |  | -2.3599 | 1.9963 |
|  |  | WT-Neuritin | -.90909 | .78467 | 1.000 |  | -3.0872 | 1.2690 |
|  |  | Tg-Control | -.09091 | .78467 | 1.000 |  | -2.2690 | 2.0872 |
| Distance020 | WT-Control | WT-Neuritin | -2.90909 | 1.28243 | .173 |  | -6.4689 | .6508 |
|  |  | Tg-Control | .63636 | 1.28243 | 1.000 |  | -2.9235 | 4.1962 |
|  |  | Tg-Neuritin | .27273 | 1.28243 | 1.000 |  | -3.2871 | 3.8326 |
|  | WT-Neuritin | WT-Control | 2.90909 | 1.28243 | .173 |  | -.6508 | 6.4689 |
|  |  | Tg-Control | 3.54545 | 1.28243 | .051 |  | -.0144 | 7.1053 |
|  |  | Tg-Neuritin | 3.18182 | 1.28243 | .104 |  | -.3780 | 6.7417 |
|  | Tg-Control | WT-Control | -.63636 | 1.28243 | 1.000 |  | -4.1962 | 2.9235 |
|  |  | WT-Neuritin | -3.54545 | 1.28243 | .051 |  | -7.1053 | .0144 |
|  |  | Tg-Neuritin | -.36364 | 1.28243 | 1.000 |  | -3.9235 | 3.1962 |
|  | Tg-Neuritin | WT-Control | -.27273 | 1.28243 | 1.000 |  | -3.8326 | 3.2871 |
|  |  | WT-Neuritin | -3.18182 | 1.28243 | .104 |  | -6.7417 | .3780 |
|  |  | Tg-Control | .36364 | 1.28243 | 1.000 |  | -3.1962 | 3.9235 |
| Distance030 | WT-Control | WT-Neuritin | -2.45455 | 1.43481 | .569 |  | -6.4374 | 1.5283 |
|  |  | Tg-Control | 1.36364 | 1.43481 | 1.000 |  | -2.6192 | 5.3465 |
|  |  | Tg-Neuritin | -.09091 | 1.43481 | 1.000 |  | -4.0737 | 3.8919 |
|  | WT-Neuritin | WT-Control | 2.45455 | 1.43481 | .569 |  | -1.5283 | 6.4374 |
|  |  | Tg-Control | 3.81818 | 1.43481 | .067 |  | -.1647 | 7.8010 |
|  |  | Tg-Neuritin | 2.36364 | 1.43481 | .644 |  | -1.6192 | 6.3465 |
|  | Tg-Control | WT-Control | -1.36364 | 1.43481 | 1.000 |  | -5.3465 | 2.6192 |
|  |  | WT-Neuritin | -3.81818 | 1.43481 | .067 |  | -7.8010 | .1647 |
|  |  | Tg-Neuritin | -1.45455 | 1.43481 | 1.000 |  | -5.4374 | 2.5283 |
|  | Tg-Neuritin | WT-Control | .09091 | 1.43481 | 1.000 |  | -3.8919 | 4.0737 |
|  |  | WT-Neuritin | -2.36364 | 1.43481 | .644 |  | -6.3465 | 1.6192 |
|  |  | Tg-Control | 1.45455 | 1.43481 | 1.000 |  | -2.5283 | 5.4374 |
| Distance040 | WT-Control | WT-Neuritin | -2.45455 | 1.68807 | .922 |  | -7.1404 | 2.2313 |
|  |  | Tg-Control | 3.90909 | 1.68807 | .155 |  | -.7768 | 8.5950 |
|  |  | Tg-Neuritin | .36364 | 1.68807 | 1.000 |  | -4.3222 | 5.0495 |
|  | WT-Neuritin | WT-Control | 2.45455 | 1.68807 | .922 |  | -2.2313 | 7.1404 |
|  |  | Tg-Control | 6.36364 | 1.68807 | .003 |  | 1.6778 | 11.0495 |
|  |  | Tg-Neuritin | 2.81818 | 1.68807 | .617 |  | -1.8677 | 7.5040 |
|  | Tg-Control | WT-Control | -3.90909 | 1.68807 | .155 |  | -8.5950 | .7768 |
|  |  | WT-Neuritin | -6.3636 | 1.68807 | .003 |  | -11.0495 | -1.6778 |
|  |  | Tg-Neuritin | -3.54545 | 1.68807 | .252 |  | -8.2313 | 1.1404 |
|  | Tg-Neuritin | WT-Control | -.36364 | 1.68807 | 1.000 |  | -5.0495 | 4.3222 |
|  |  | WT-Neuritin | -2.81818 | 1.68807 | .617 |  | -7.5040 | 1.8677 |
|  |  | Tg-Control | 3.54545 | 1.68807 | .252 |  | -1.1404 | 8.2313 |
| Distance050 | WT-Control | WT-Neuritin | -2.90909 | 1.87546 | .772 |  | -8.1151 | 2.2969 |
|  |  | Tg-Control | 6 | 1.87546 | .016 | * | .7940 | 11.2060 |
|  |  | Tg-Neuritin | .81818 | 1.87546 | 1.000 |  | -4.3878 | 6.0242 |
|  | WT-Neuritin | WT-Control | 2.90909 | 1.87546 | .772 |  | -2.2969 | 8.1151 |
|  |  | Tg-Control | 8.90909 | 1.87546 | .000 |  | 3.7031 | 14.1151 |
|  |  | Tg-Neuritin | 3.72727 | 1.87546 | .323 |  | -1.4788 | 8.9333 |
|  | Tg-Control | WT-Control | -6 | 1.87546 | .016 | * | -11.2060 | -.7940 |
|  |  | WT-Neuritin | -8.90909 | 1.87546 | .000 |  | -14.1151 | -3.7031 |
|  |  | Tg-Neuritin | -5.18182 | 1.87546 | .052 |  | -10.3878 | .0242 |
|  | Tg-Neuritin | WT-Control | -.81818 | 1.87546 | 1.000 |  | -6.0242 | 4.3878 |
|  |  | WT-Neuritin | -3.72727 | 1.87546 | .323 |  | -8.9333 | 1.4788 |
|  |  | Tg-Control | 5.18182 | 1.87546 | .052 |  | -.0242 | 10.3878 |
| Distance060 | WT-Control | WT-Neuritin | -5.09091 | 2.14418 | .135 |  | -11.0429 | .8611 |
|  |  | Tg-Control | 8.81818 | 2.14418 | .001 | ** | 2.8662 | 14.7701 |
|  |  | Tg-Neuritin | .72727 | 2.14418 | 1.000 |  | -5.2247 | 6.6792 |
|  | WT-Neuritin | WT-Control | 5.09091 | 2.14418 | .135 |  | -.8611 | 11.0429 |
|  |  | Tg-Control | 13.90909 | 2.14418 | .000 |  | 7.9571 | 19.8611 |
|  |  | Tg-Neuritin | 5.81818 | 2.14418 | .059 |  | -.1338 | 11.7701 |
|  | Tg-Control | WT-Control | -8.81818 | 2.14418 | .001 | ** | -14.7701 | -2.8662 |
|  |  | WT-Neuritin | -13.90909 | 2.14418 | .000 |  | -19.8611 | -7.9571 |
|  |  | Tg-Neuritin | -8.09091 | 2.14418 | .003 | ‡‡ | -14.0429 | -2.1389 |
|  | Tg-Neuritin | WT-Control | -.72727 | 2.14418 | 1.000 |  | -6.6792 | 5.2247 |
|  |  | WT-Neuritin | -5.81818 | 2.14418 | .059 |  | -11.7701 | .1338 |
|  |  | Tg-Control | 8.09091 | 2.14418 | .003 | ‡‡ | 2.1389 | 14.0429 |
| Distance070 | WT-Control | WT-Neuritin | -4.72727 | 2.05120 | .159 |  | -10.4211 | .9666 |
|  |  | Tg-Control | 11.54545 | 2.05120 | .000 | *** | 5.8516 | 17.2393 |
|  |  | Tg-Neuritin | 3.81818 | 2.05120 | .420 |  | -1.8757 | 9.5121 |
|  | WT-Neuritin | WT-Control | 4.72727 | 2.05120 | .159 |  | -.9666 | 10.4211 |
|  |  | Tg-Control | 16.27273 | 2.05120 | .000 |  | 10.5789 | 21.9666 |
|  |  | Tg-Neuritin | 8.54545 | 2.05120 | .001 |  | 2.8516 | 14.2393 |
|  | Tg-Control | WT-Control | -11.54545 | 2.05120 | .000 | *** | -17.2393 | -5.8516 |
|  |  | WT-Neuritin | -16.27273 | 2.05120 | .000 |  | -21.9666 | -10.5789 |
|  |  | Tg-Neuritin | -7.72727 | 2.05120 | .003 | ‡‡ | -13.4211 | -2.0334 |
|  | Tg-Neuritin | WT-Control | -3.81818 | 2.05120 | .420 |  | -9.5121 | 1.8757 |
|  |  | WT-Neuritin | -8.54545 | 2.05120 | .001 |  | -14.2393 | -2.8516 |
|  |  | Tg-Control | 7.72727 | 2.05120 | .003 | ‡‡ | 2.0334 | 13.4211 |
| Distance080 | WT-Control | WT-Neuritin | -5.54545 | 2.44932 | .174 |  | -12.3444 | 1.2535 |
|  |  | Tg-Control | 12.54545 | 2.44932 | .000 | *** | 5.7465 | 19.3444 |
|  |  | Tg-Neuritin | 4.09091 | 2.44932 | .616 |  | -2.7081 | 10.8899 |
|  | WT-Neuritin | WT-Control | 5.54545 | 2.44932 | .174 |  | -1.2535 | 12.3444 |
|  |  | Tg-Control | 18.09091 | 2.44932 | .000 |  | 11.2919 | 24.8899 |
|  |  | Tg-Neuritin | 9.63636 | 2.44932 | .002 |  | 2.8374 | 16.4354 |
|  | Tg-Control | WT-Control | -12.54545 | 2.44932 | .000 | *** | -19.3444 | -5.7465 |
|  |  | WT-Neuritin | -18.09091 | 2.44932 | .000 |  | -24.8899 | -11.2919 |
|  |  | Tg-Neuritin | -8.45455 | 2.44932 | .008 | ‡‡ | -15.2535 | -1.6556 |
|  | Tg-Neuritin | WT-Control | -4.09091 | 2.44932 | .616 |  | -10.8899 | 2.7081 |
|  |  | WT-Neuritin | -9.63636 | 2.44932 | .002 |  | -16.4354 | -2.8374 |
|  |  | Tg-Control | 8.45455 | 2.44932 | .008 | ‡‡ | 1.6556 | 15.2535 |
| Distance090 | WT-Control | WT-Neuritin | -3.00000 | 2.55534 | 1.000 |  | -10.0933 | 4.0933 |
|  |  | Tg-Control | 16.54545 | 2.55534 | .000 | *** | 9.4522 | 23.6387 |
|  |  | Tg-Neuritin | 7.00000 | 2.55534 | .055 |  | -.0933 | 14.0933 |
|  | WT-Neuritin | WT-Control | 3.00000 | 2.55534 | 1.000 |  | -4.0933 | 10.0933 |
|  |  | Tg-Control | 19.5454 | 2.55534 | .000 |  | 12.4522 | 26.6387 |
|  |  | Tg-Neuritin | 10 | 2.55534 | .002 |  | 2.9067 | 17.0933 |
|  | Tg-Control | WT-Control | -16.54545 | 2.55534 | .000 | *** | -23.6387 | -9.4522 |
|  |  | WT-Neuritin | -19.54545 | 2.55534 | .000 |  | -26.6387 | -12.4522 |
|  |  | Tg-Neuritin | -9.54545 | 2.55534 | .004 | ‡‡ | -16.6387 | -2.4522 |
|  | Tg-Neuritin | WT-Control | -7.00000 | 2.55534 | .055 |  | -14.0933 | .0933 |
|  |  | WT-Neuritin | -10.00000^*^ | 2.55534 | .002 |  | -17.0933 | -2.9067 |
|  |  | Tg-Control | 9.54545 | 2.55534 | .004 | ‡‡ | 2.4522 | 16.6387 |
| Distance100 | WT-Control | WT-Neuritin | -2.54545 | 2.97052 | 1.000 |  | -10.7912 | 5.7003 |
|  |  | Tg-Control | 13.36364 | 2.97052 | .000 | *** | 5.1179 | 21.6094 |
|  |  | Tg-Neuritin | 5.63636 | 2.97052 | .390 |  | -2.6094 | 13.8821 |
|  | WT-Neuritin | WT-Control | 2.54545 | 2.97052 | 1.000 |  | -5.7003 | 10.7912 |
|  |  | Tg-Control | 15.90909 | 2.97052 | .000 |  | 7.6633 | 24.1548 |
|  |  | Tg-Neuritin | 8.18182 | 2.97052 | .053 |  | -.0639 | 16.4276 |
|  | Tg-Control | WT-Control | -13.36364 | 2.97052 | .000 | *** | -21.6094 | -5.1179 |
|  |  | WT-Neuritin | -15.90909 | 2.97052 | .000 |  | -24.1548 | -7.6633 |
|  |  | Tg-Neuritin | -7.72727 | 2.97052 | .078 |  | -15.9730 | .5185 |
|  | Tg-Neuritin | WT-Control | -5.63636 | 2.97052 | .390 |  | -13.8821 | 2.6094 |
|  |  | WT-Neuritin | -8.18182 | 2.97052 | .053 |  | -16.4276 | .0639 |
|  |  | Tg-Control | 7.72727 | 2.97052 | .078 |  | -.5185 | 15.9730 |
| Distance110 | WT-Control | WT-Neuritin | -1.63636 | 3.22272 | 1.000 |  | -10.5822 | 7.3095 |
|  |  | Tg-Control | 15.63636 | 3.22272 | .000 | *** | 6.6905 | 24.5822 |
|  |  | Tg-Neuritin | 6.36364 | 3.22272 | .331 |  | -2.5822 | 15.3095 |
|  | WT-Neuritin | WT-Control | 1.63636 | 3.22272 | 1.000 |  | -7.3095 | 10.5822 |
|  |  | Tg-Control | 17.27273 | 3.22272 | .000 |  | 8.3269 | 26.2186 |
|  |  | Tg-Neuritin | 8.00000 | 3.22272 | .104 |  | -.9459 | 16.9459 |
|  | Tg-Control | WT-Control | -15.63636 | 3.22272 | .000 | *** | -24.5822 | -6.6905 |
|  |  | WT-Neuritin | -17.27273 | 3.22272 | .000 |  | -26.2186 | -8.3269 |
|  |  | Tg-Neuritin | -9.27273 | 3.22272 | .038 | ‡ | -18.2186 | -.3269 |
|  | Tg-Neuritin | WT-Control | -6.36364 | 3.22272 | .331 |  | -15.3095 | 2.5822 |
|  |  | WT-Neuritin | -8.00000 | 3.22272 | .104 |  | -16.9459 | .9459 |
|  |  | Tg-Control | 9.27273 | 3.22272 | .038 | ‡ | .3269 | 18.2186 |
| Distance120 | WT-Control | WT-Neuritin | .45455 | 3.25043 | 1.000 |  | -8.5682 | 9.4773 |
|  |  | Tg-Control | 14.09091 | 3.25043 | .001 | *** | 5.0682 | 23.1137 |
|  |  | Tg-Neuritin | 4.36364 | 3.25043 | 1.000 |  | -4.6591 | 13.3864 |
|  | WT-Neuritin | WT-Control | -.45455 | 3.25043 | 1.000 |  | -9.4773 | 8.5682 |
|  |  | Tg-Control | 13.63636 | 3.25043 | .001 |  | 4.6136 | 22.6591 |
|  |  | Tg-Neuritin | 3.90909 | 3.25043 | 1.000 |  | -5.1137 | 12.9318 |
|  | Tg-Control | WT-Control | -14.09091 | 3.25043 | .001 | *** | -23.1137 | -5.0682 |
|  |  | WT-Neuritin | -13.63636 | 3.25043 | .001 |  | -22.6591 | -4.6136 |
|  |  | Tg-Neuritin | -9.72727 | 3.25043 | .028 | ‡ | -18.7500 | -.7045 |
|  | Tg-Neuritin | WT-Control | -4.36364 | 3.25043 | 1.000 |  | -13.3864 | 4.6591 |
|  |  | WT-Neuritin | -3.90909 | 3.25043 | 1.000 |  | -12.9318 | 5.1137 |
|  |  | Tg-Control | 9.72727^*^ | 3.25043 | .028 | ‡ | .7045 | 18.7500 |
| Total | WT-Control | WT-Neuritin | -33.54545 | 19.03735 | .514 |  | -86.3906 | 19.2997 |
|  |  | Tg-Control | 104.54545^*^ | 19.03735 | .000 | *** | 51.7003 | 157.3906 |
|  |  | Tg-Neuritin | 33.54545 | 19.03735 | .514 |  | -19.2997 | 86.3906 |
|  | WT-Neuritin | WT-Control | 33.54545 | 19.03735 | .514 |  | -19.2997 | 86.3906 |
|  |  | Tg-Control | 138.09091 | 19.03735 | .000 |  | 85.2458 | 190.9360 |
|  |  | Tg-Neuritin | 67.09091 | 19.03735 | .006 |  | 14.2458 | 119.9360 |
|  | Tg-Control | WT-Control | -104.5454 | 19.03735 | .000 | *** | -157.3906 | -51.7003 |
|  |  | WT-Neuritin | -138.09091 | 19.03735 | .000 |  | -190.9360 | -85.2458 |
|  |  | Tg-Neuritin | -71 | 19.03735 | .004 | ‡‡ | -123.8451 | -18.1549 |
|  | Tg-Neuritin | WT-Control | -33.54545 | 19.03735 | .514 |  | -86.3906 | 19.2997 |
|  |  | WT-Neuritin | -67.09091 | 19.03735 | .006 |  | -119.9360 | -14.2458 |
|  |  | Tg-Control | 71 | 19.03735 | .004 | ‡‡ | 18.1549 | 123.8451 |
| Statistical significance between WT-Control vs. Tg-Control is expressed as **P* < 0.05; ***P* < 0.01; | | | | | | | | |
| ****P* < 0.001 and the comparison between Tg-Control vs. Tg-Neuritin is expressed | | | | | | | |  |
| as ‡*P* < 0.05; ‡‡*P* < 0.01. | | |  |  |  |  |  |  |
