# Supplementary figures and images for: Neuritin can normalize neural deficits of Alzheimer's disease
Source: Cell Death Dis. 2014 Nov 13;5(11):e1523–. doi: 10.1038/cddis.2014.478 (PMC4260736; doi:10.1038/cddis.2014.478)

**Supplementary Figure 1.**

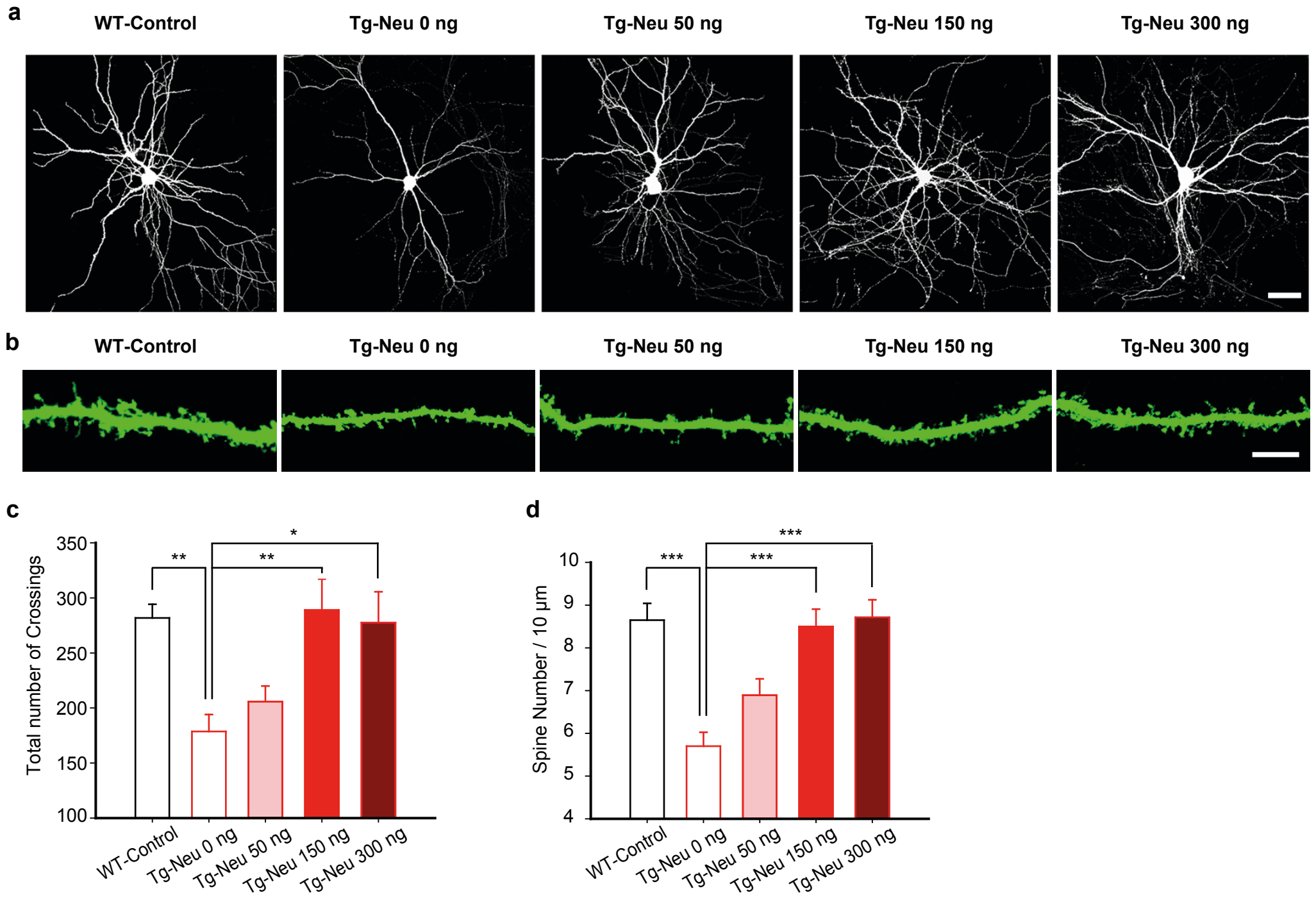

Supplement: Supplementary Information [file cddis2014478x2.pdf]
